# Evaluation of factors affecting sexual dysfunction in female patients with diabetes mellitus

**DOI:** 10.20945/2359-3997000000238

**Published:** 2020-03-30

**Authors:** Mustafa Gürkan Yenice, Yavuz Onur Danacıoğlu, Meral Mert, Pınar Karakaya, Kamil Gokhan Seker, Fatih Akkaş, Abdulmuttalip Şimşek, Selçuk Şahin, Ali Ihsan Taşçı

**Affiliations:** 1 Clinic of Urology University of Health Sciences Bakırköy Dr. Sadi Konuk Training and Research Hospital Istanbul Turkey Clinic of Urology, University of Health Sciences, Bakırköy Dr. Sadi Konuk Training and Research Hospital, Istanbul, Turkey; 2 Department of Endocrinology University of Health Sciences Bakırköy Dr. Sadi Konuk Training and Research Hospital Istanbul Turkey Department of Endocrinology, University of Health Sciences, Bakırköy Dr. Sadi Konuk Training and Research Hospital, Istanbul, Turkey; 3 Clinic of Urology University of Health Sciences Bakırköy Dr. Sadi Konuk Training and Research Hospital Istanbul Turkey Clinic of Urology, University of Health Sciences, Bakırköy Dr. Sadi Konuk Training and Research Hospital, Istanbul, Turkey

**Keywords:** Arylesterase, diabetes mellitus, endocrine disorder, female sexual dysfunction, paraoxonase-1

## Abstract

**Objective:**

Our objective in this study was to evaluate the factors predicting female sexual dysfunction (FSD) in patients with diabetes mellitus (DM).

**Subjects and methods:**

The study included 149 women with DM. Sexual function was evaluated with the Female Sexual Function Index (FSFI) questionnaire, in which total scores under 26.55 characterized the occurrence of FSD (Group 1 > 26.55, Group 2 < 26.55). We recorded the patients’ demographic, metabolic, and hormonal data. Ophthalmologic, neurologic, and renal complications were also evaluated. The antioxidant status of the patients in both groups was determined by measuring the activity of the enzymes paraoxonase-1 (PON-1) and arylesterase (ARE).

**Results:**

Based on the FSFI scores, 60 patients were allocated to Group 1 (26.6 ± 12.3) and 89 to Group 2 (22.6 ± 9.5). Group 2 compared with Group 1 had significantly (p < 0.05) higher mean concentrations of glycated hemoglobin (HbA1c), glucose, triglycerides, and insulin, along with higher rates of metformin use, smoking, retinopathy, and nephropathy. The mean serum ARE concentrations were significantly lower in Group 2 compared with Group 1 (p = 0.000), but the mean serum PON-1 concentrations were similar between both groups (p = 0.218). On multivariable regression analysis, age, ARE activity, Beck Depression Inventory (BDI) score, and menopause were significant independent predictors of FSD (p < 0.05).

**Conclusions:**

In this study, we evaluated the predictive factors determining FSD caused by DM. Despite the significant results found in our study, future randomized controlled studies with a long follow-up and a larger number of patients are required to determine how DM affects FSD.

## INTRODUCTION

Diabetes mellitus (DM) is one of the most common noninfectious progressive and chronic diseases worldwide, constituting an important current health problem (1). The prevalence of type 2 DM is increasing at a fast pace in developed and developing countries due to lifestyle changes. The 2017 International Diabetes Federation (IDF) DM map predicted that 425 million people worldwide had type 2 DM, and that this number will reach nearly 630 million with a 48% increase by 2045 (2). Microvascular and macrovascular complications of DM lead to loss of function in many organs and systems.

In the long term, DM causes complications such as chronic renal disease, peripheral neuropathy, retinopathy, and cardiovascular disorders (3). Sexual dysfunction may affect both sexes due to vascular, psychogenic, and neurogenic disorders caused by DM (4). Until recently, sexual dysfunction in patients with DM was attributed to psychogenic factors (5). Due to cultural and ethnic causes, sexual dysfunction caused by DM can be overlooked in women. In published studies, sexual function disorder is more often investigated in men and considered less important in women. Thus, the etiology of DM-associated female sexual dysfunction (FSD) and its risk factors are still controversial. The literature reports FSD as occurring more frequently in postmenopausal women, at a prevalence of 40-60% (6). Also, a higher prevalence of FSD is seen in women with both types (1 and 2) DM compared with nondiabetic normal women (7,8).

Multifactorial causes due to hyperglycemia, infection, vascular, neurogenic, and psychogenic factors lead to sexual dysfunction in women with diabetes. Systemic adverse events caused by diabetes lead to vaginal and clitoral hemodynamic deterioration, lubrication problems, decreased genital muscle activity, and loss of sensitivity in the genital area (3,9). Diabetes duration, poor glycemic control, advanced age, presence of complications, low-quality partner relationships, and poor cognitive adjustment have been previously defined as predictors of FSD (8). Also, the relationship between FSD and obesity, metabolic syndrome, and microvascular complications has been emphasized in recent studies (6).

In the present study, our objective was to determine psychogenic and organic predictive factors causing FSD in women with DM presenting with symptoms of sexual dysfunction.

## SUBJECTS AND METHODS

The study protocol was approved by the local ethics committee at Bakirkoy Dr. Sadi Konuk Training and Research Hospital, and the study was conducted according to the Declaration of Helsinki. All subjects were informed about the study protocol, and written consents were obtained. This prospective clinical trial was conducted between January and December 2018.

A total of 149 women with diabetes and prediabetes admitted to our outpatient diabetes clinic were enrolled in the study. All participants met the following inclusion criteria: minimum age of 18 years, no concomitant pathologies, no medication use (except for antidiabetic agents), no hormonal abnormalities (including follicle-stimulating hormone, luteinizing hormone, prolactin, estradiol, and androgens), heterosexual status, absence of sexual disorder in the male partner, and steady relationship for at least 1 year. The exclusion criteria were pregnancy or post-partum, chronic illness, diagnosis of psychiatric disorder, and use of hormonal contraception.

The Female Sexual Function Index (FSFI) questionnaire was used to evaluate sexual functioning, and the Beck Depression Inventory (BDI) was used to evaluate depression severity. Both questionnaires were self-filled by each patient. The FSFI is a self-reported form that includes 19 items measuring female sexual functioning. This questionnaire addresses desire, arousal, lubrication, orgasm, satisfaction, and pain under six main headings. Total FSFI scores below 26 categorized FSD in the present study (10). The BDI is a self-evaluation scale with 21 items applied to measure physical, emotional, and cognitive symptoms present in depression. Total BDI scores were regarded as indicating mild depression when between 10-18, moderate depression when between 19-29, and severe depression when > 30 (11). Based on FSFI scores, the patients were categorized into Group 1 (scores > 26.55) and Group 2 (scores < 26.55).

Each patient underwent a physical and genitourinary examination. Age, body mass index (BMI), parity, comorbid disorders (hypertension), use of oral antidiabetic agent (metformin) and insulin, smoking and alcohol use, and menopause duration were recorded for each patient.

Blood samples were collected after 12-hour overnight fasting for measurement of routine biochemical tests, lipid profile, and levels of glycated hemoglobin (HbA1c), insulin, creatinine, thyroid-stimulating hormone (TSH), free thyroxine (FT4), and free triiodothyronine (FT3). Nephropathy, retinopathy, polyneuropathy, and hypertension were diagnosed based on medical history, physical examination, and laboratory findings. Patients with a mean blood pressure level ≥ 140/90 mmHg and those on antihypertensive medications were classified as hypertensive. Eye complications were defined as the presence of cataract or any grade of diabetic retinopathy or maculopathy on dilated eye examination. Renal complications included microalbuminuria or macroalbuminuria. The Diabetic Neuropathy Index, which detects somatic and autonomic neuropathy, was used to assess the presence of diabetic neuropathy.

A novel method was used to determine paraoxonase-1 (PON-1) and arylesterase (ARE) activity levels (12). Briefly, the paraoxon hydrolysis (diethyl-p-nitrophenylphosphate) rate was determined by measuring the increase in absorbance at 412 nm and 25 °C. PON-1 activity is expressed as units per liter (U/L) of serum. Using spectrophotometry, ARE activity was measured using phenylacetate as a substrate. The reaction was started by the addition of the serum and measured by the absorbance increase at 270 nm. Enzymatic activity was calculated from the molar absorptivity coefficient of the produced phenol. One unit of ARE activity was defined as 1 μmol of phenol generated per minute under the defined assay conditions and is also expressed as U/L (13).

### Statistical analysis

The data are described as rates and mean, standard deviation, median, minimum and maximum, and frequency values. The Kolmogorov-Smirnov test was used to analyze the distribution of the variables. Student’s* t* test and Mann-Whitney U test were used to analyze independent quantitative data, while the chi-square test was used to analyze independent qualitative data. The effect level was investigated using univariate and multivariate logistic regression. The SPSS 22.0 statistics software (IBM Corp., Armonk, NY, USA) was used for the analyses.

## RESULTS

The baseline characteristics of all 149 women are summarized in Table 1. Based on the FSFI scores, 60 patients were allocated to Group 1 and 89 patients to Group 2. The mean total FSFI score was significantly lower in Group 2 (22.6 ± 9.5) compared with Group 1 (26.6 ± 12.3; p = 0.012). The mean diabetes duration was 11.1 ± 6.6 years, the mean glucose value was 147.3 ± 60.4 mg/dL, and the mean HbA1c value was 7.2 ± 1.8%. The mean age of the women in Groups 1 and 2 was 50.1 ± 13.0 years and 53.2 ± 13.2 years, respectively. The mean ages of the participants in both groups were similar (p = 0.154). Mean BMI was also similar between Groups 1 and 2 (31.7 ± 5.3 kg/m^2^ and 29.6 ± 5.8 kg/m^2^, respectively; p = 0.030). The mean BDI score was higher in Group 2 (15.9 ± 8.2) compared with Group 1 (10.1 ± 7.3; p = 0.000). The mean TSH concentration was also significantly lower in Group 2 compared with Group 1 (2.4 ± 1.8 mU/L versus 4.0 ± 2.8 mU/L, respectively; p = 0.07).

**Table 1:** Demographic and clinical data of the participants

	Min	Max	Median	Mean ± SD/ n (%)
Age (years)	19.0	83.0	52.0	52.0 ± 13.2
BMI (kg/m^2^)	19.0	48.0	30.0	30.5 ± 5.7
DM duration (years)	1.0	36.0	9.0	11.1 ± 6.6
Parity (n)	0.0	13.0	2.0	2.4 ± 2.3
HbA1c (%)	2.0	15.8	6.8	7.2 ± 1.8
Glucose (mg/dL)	82.0	389.0	126.0	147.3 ± 60.4
Creatinine (mg/dL)	0.2	37.0	0.6	0.9 ± 3.0
TSH (mU/L)	0.0	10.3	2.0	3.0 ± 2.4
FT4 (mU/L)	0.1	1.9	0.9	0.9 ± 0.2
Hgb (g/dL)	7.4	16.0	12.5	12.4 ± 1.3
HDL (mg/dL)	24.0	87.0	48.0	49.0 ± 10.3
LDL (mg/dL)	11.0	275.0	125.5	127.0 ± 38.0
Total cholesterol (mg/dL)	127.0	325.0	198.0	204.9 ± 42.1
Triglycerides (mg/dL)	48.0	477.0	135.0	152.4 ± 74.4
PON-1 (U/L)	46.6	262.0	117.2	121.5 ± 38.7
ARE (U/L)	90.8	279.2	149.0	152.2 ± 41.5
Insulin use				51 34.0%
Metformin use				59 39.3%
Smoking				14 9.3%
Alcohol use				14 9.3%
Menopause				65 43.3%
Duration of menopause	2.0	40.0	12.0	12.7 ± 7.7
Retinopathy				14 9.3%
Neuropathy				27 18.0%
Nephropathy				9 6.0%
Hypertension				71 47.3%
BDI	2.0	40.0	11.5	13.6 ± 8.3
FSFI	2.0	76.0	26.5	24.2 ± 10.8
Sexual dysfunction				70 46.7%

Min-Max: minimum-maximum; SD: Standard deviation; BMI: body mass index; DM: diabetes mellitus; HbA1c: glycated hemoglobin; TSH: thyroid-stimulating hormone; FT4: free thyroxine; Hgb: hemoglobin; HDL: high-density lipoprotein; LDL: low-density lipoprotein; PON-1: paraoxonase-1; ARE: arylesterase; BDI: Beck Depression Inventory; FSFI: Female Sexual Functioning Index.

The mean concentrations of HbA1c, glucose, triglycerides, and insulin, as well as the rates of metformin use, smoking, retinopathy, and nephropathy, were significantly higher in Group 2 than Group 1 (p < 0.05). Groups 1 and 2 showed no differences in regard to levels of creatine, FT4, hemoglobin, high-density lipoprotein (HDL), low-density lipoprotein (LDL), and total cholesterol, or in terms of duration of alcohol use and rates and duration of menopause and hypertension (p > 0.05). The mean serum ARE concentrations were lower in Group 2 (138.7 ± 33.0 U/L) compared with Group 1 (172.7 ± 44.8; p = 0.000), but no significant difference was observed in mean serum PON-1 concentrations between both groups (118.1 ± 35.7 versus 126.7±42.7, respectively, p = 0.218) (Table 2).

**Table 2:** Comparison of the general characteristics and biochemical studies

	Group 1		Group 2		p value

	Mean ± SD/n (%)	Median	Mean ± SD/n (%)	Median	
Age (years)	50.1 ± 13.0	50.0	53.2 ± 13.2	54.5	0.154 ^t^
BMI (kg/m^2^)	31.7 ± 5.3	30.5	29.6 ± 5.8	29.0	**0.030** ^m^
Parity (n)	2.7 ± 2.3	2.0	2.3 ± 2.4	2.0	0.148 ^m^
HbA1c (%)	6.4 ± 1.0	6.2	7.7 ± 2.0	7.4	**0.000** ^m^
Glucose (mg/dL)	111.5 ± 14.2	112.0	171.1 ± 67.4	148.5	**0.000** ^m^
Creatinine (mg/dL)	1.2 ± 4.7	0.6	0.7 ± 0.5	0.6	0.061 ^m^
TSH (mU/L)	4.0 ± 2.8	3.2	2.4 ± 1.8	1.9	**0.007** ^m^
FT4 (mU/L)	0.9 ± 0.2	0.9	1.0 ± 0.2	1.0	0.059 ^m^
Hgb (g/dL)	12.3 ± 0.9	12.5	12.4 ± 1.4	12.6	0.574 ^m^
HDL (mg/dL)	48.5 ± 9.6	48.0	49.3 ± 10.8	49.5	0.864 ^m^
LDL (mg/dL)	130.0 ± 33.3	126.5	125.0 ± 40.8	121.5	0.482 ^m^
Total cholesterol (mg/dL)	205.6 ± 37.8	196.5	204.4 ± 45.0	200.0	0.168 ^m^
Triglycerides (mg/dL)	125.2 ± 49.4	114.0	170.5 ± 82.5	153.0	**0.000** ^m^
PON-1 (U/L)	126.7 ± 42.7	122.6	118.1 ± 35.7	109.9	0.218 ^m^
ARE (U/L)	172.7 ± 44.8	185.9	138.7 ± 33.0	130.5	**0.000** ^m^
Insulin use	0 0.0%		51 56.7%		**0.000** ^X2^
Metformin use	0 0.0%		59 65.6%		**0.000** ^X2^
Smoking	0 0.0%		14 15.6%		**0.001** ^X2^
Alcohol use	4 6.7%		10 11.1%		0.359 ^X2^
Menopause	26 43.3%		39 43.3%		1.000 ^X2^
Menopause duration	11.9 ± 8.0	12.0	13.2 ± 7.5	14.0	0.321 ^m^
Retinopathy	0 0.0%		14 15.6%		**0.001** ^X2^
Neuropathy	0 0.0%		27 30.0%		**0.000** ^X2^
Nephropathy	0 0.0%		9 10.0%		**0.012** ^X2^
Hypertension	23 38.3%		48 53.3%		0.071 ^X2^
BDI	10.1 ± 7.3	8.0	15.9 ± 8.2	14.0	**0.000 ** ^m^
FSFI	26.6 ± 12.3	29.0	22.6 ± 9.5	24.5	**0.012** ^m^
Sexual dysfunction	20 33.3%		50 55.6%		**0.008 ** ^X2^

^t ^Student’s t test; ^m^ Mann-Withney U test; ^X2^chi-square test. Abbreviations – Min-Max: minimummaximum; SD: Standard deviation; BMI: body mass index; DM: diabetes mellitus; HbA1c: glycated hemoglobin; TSH: thyroid-stimulating hormone; FT4: free thyroxine; Hgb: hemoglobin; HDL: highdensity lipoprotein; LDL: low-density lipoprotein; PON-1: paraoxonase-1; ARE: arylesterase; BDI: Beck Depression Inventory; FSFI: Female Sexual Functioning Index.

Age; parity; values of HbA1c, creatinine, TSH, and triglycerides; ARE activity; BDI scores; and rates of insulin use, menopause, nephropathy, and hypertension emerged as significant predictors of diabetic FSD on univariate regression analysis (p ˂ 0.05). On multivariate regression analysis, age, ARE activity, BDI, and menopause remained as significant independent predictors of FSD (p < 0.05) (Table 3).

**Table 3:** Univariate and multivariate logistic regression analyses

	Univariate analysis			Multivariate analysis			
	
	OR	95% CI	p value	OR	95%	CI	p value
Age (years)	1.19	1.13-1.26	***0.000***	1.27	1.15	1.41	***0.000***
BMI (kg/m^2^)	0.97	0.92-1.03	0.313				
Parity (n)	1.19	1.02-1.38	***0.023***				
HbA1c (%)	1.27	1.04-1.55	***0.019***				
Glucose (mg/dL)	1.00	1.00-1.01	0.115				
Creatinine (mg/dL)	82.97	8.35->100	***0.000***				
TSH (mU/L)	0.84	0.72-0.97	***0.019***				
FT4 (mU/L)	1.63	0.38-7.01	0.509				
Hgb (g/dL)	1.04	0.81-1.34	0.759				
Total cholesterol (mg/dL)	1.00	0.99-1.01	0.513				
HDL (mg/dL)	0.99	0.96-1.02	0.474				
LDL (mg/dL)	1.00	0.99-1.01	0.897				
Triglycerides (mg/dL)	1.01	1.00-1.01	***0.003***				
PON-1 (U/L)	0.99	0.98-1.00	0.107				
ARE (U/L)	0.99	0.98-1.00	***0.023***	0.99	0.97	1.00	***0.018***
BDI	1.15	1.09-1.22	***0.000***	1.11	1.04	1.19	***0.002***
Insulin use	3.07	1.52-6.21	***0.002***				
Metformin use	1.85	0.95-3.59	0.068				
Smoking	0.84	0.28-2.56	0.764				
Alcohol use	1.59	0.52-4.83	0.412				
Menopause	4.75	2.37-9.53	***0.000***	0.21	0.05	0.90	***0.035***
Retinopathy	3.17	0.95-10.60	0.061				
Neuropathy	2.25	0.95-5.30	0.065				
Nephropathy	10.19	1.24-83.68	***0.031***				
Hypertension	4.77	2.39-9.52	***0.000***				

OR: odds ratio; CI: confidence interval; BMI: body mass index; HbA1c: glycated hemoglobin; TSH: thyroid-stimulating hormone; FT4: free thyroxine; Hgb: hemoglobin; HDL: high-density lipoprotein; LDL: low-density lipoprotein; PON-1: paraoxonase-1; ARE: arylesterase; BDI: Beck Depression Inventory.

## DISCUSSION

The World Health Organization (WHO) declared human sexuality to be part of health quality and well-being in 1974. In women, sexual function depends on different physiological circumstances such as vaginal hemodynamics and neurologic innervation, and the activity of genital and pelvic structures (3,14). Sexual disorders in patients with DM are related to psychological problems occurring due to medications or vascular and neuroendocrine complications of the disease (15).

Sexual dysfunction in female patients with DM occurs both due to decreased clitoral blood flow related to deterioration in the hypogastric-vaginal/clitoral arterial bed, which is part of vascular impairment and concurring peripheral neuropathy in DM. Despite this information, the number of studies determining the predisposing factors causing sexual dysfunction in women with diabetes is limited, and these studies generally have an inadequate number of patients and concentrate on specific factors (2,16). We hypothesized that sexual dysfunction was related to organic and psychogenic causes in women with diabetes. Thus, our study investigated the psychogenic and underlying organic causes of FSD in a large patient population.

In our study, BDI scores were higher in patients with sexual dysfunction with low FSFI scores. Studies have shown that many psychological diseases, especially depression, cause sexual dysfunction in patients with diabetes. Both BDI scores and rates of sexual dysfunction have been reported to be higher in diabetic patients with complications compared with those without complications (17). BDI was shown to be an independent indicator of FSD in patients with DM in our study. Apart from psychogenic factors, organic causes occurring due to end-organ damage caused by diabetes also affect the sexual quality of life in women (18). Sexual activity and sexual satisfaction decrease in the presence of end-organ complications such as retinopathy, neuropathy, nephropathy, and heart disease in women with diabetes (19). In our study, the rates of nephropathy, neuropathy, and retinopathy were higher in patients with sexual dysfunction compared with those without this complication.

Blood vessel damage and clitoral nerve and autonomic nervous system degeneration are common in the genital organs of patients with diabetes, resulting in arousal and lubrication dysfunction (7,15). In our study, patients with sexual dysfunction had higher triglycerides values and poor glycemic control. Low sexual desire and arousal have been reported in menopausal patients with metabolic syndrome and poor lipid profile in previous studies (5,20,21). Several studies have also reported older age as a cause of sexual dysfunction in women (22,23). In our study, we found that menopause and older age were independent indicators of sexual dysfunction based on multivariate analysis.

Vascular damage due to DM is one of the main causes of sexual dysfunction in both sexes. Increased lipids, glucose intolerance, and presence of metabolic syndrome induce vascular damage by potentializing the occurrence of atherosclerosis in these patients. One of the most important indicators of atherosclerosis in patients with types 1 and 2 DM and metabolic syndrome is the occurrence of decreased PON and ARE enzyme activity (24,25). PON is an ester hydrolase enzyme that can hydrolyze paraoxon, which is a strong inhibitor of cholinesterases. PON-1, PON-2, and PON-3 belong to a subgroup of enzymes in the *PON* gene family. Low serum PON-1 activity is associated with hyperlipidemia, type 1 DM, coronary artery disease, chronic kidney failure, rheumatoid arthritis, metabolic syndrome, uremia, and thyroid dysfunction (26).

PON-1 and ARE are esterase group enzymes encoded by the same gene and with similar active centers. PON-1 is an enzyme with three known main activities as paraoxonase, arylesterase, and dyazoxonase (25,27,28). PON-1 and ARE contribute to the protective effect of HDL against atherosclerosis. A study by Ciftci and cols. observed a negative correlation between PON-1 activity and erectile dysfunction (ED), along with a correlation between PON-1 activity and HDL levels, while LDL levels were higher in the ED group compared with the control group (29). A study by Aldemir and cols. reported lower PON-1 and ARE activity in patients with ED compared with controls (28). In our study, PON-1 activity was lower in patients with FSD, but the difference was not statistically significant. In contrast, ARE activity was significantly lower in patients with FSD compared with those without FSD and emerged as an independent indicator of sexual activity in female patients with DM. Although the connection between PON and ARE activity and different diseases has been shown in many studies in literature, our study is the first to demonstrate the connection of FSD in women with DM with activity of the enzymes PON and ARE, both of which have antioxidant properties.

Our study has some limitations to take into consideration. The main one is the cross-sectional design. Also, the long-term effect of diabetes and related complications on sexual function was not investigated in our patients. The lack of data on lubrication and orgasm, both of which correlate with sexual desire in these patients and reflect vascular and neurologic deficit, hinders the presentation of more objective data. The sexual function of the patients in our study was considered to be normal based on the questions asked, but in our opinion, variations in sexual function between the partners may affect the patients’ FSFI scores.

In conclusion, we showed that older age, ARE activity, BDI score, and menopause are independent risk factors for FSD in women with DM. The finding of diabetes causing sexual dysfunction due to psychogenic and organic causes is evidence that these patients should undergo a multidisciplinary evaluation. Sexual dysfunction is commonly neglected in patients with diabetes and should be investigated to improve their quality of life with treatment. In this study, we attempted to present the FSD factors that affect pathogenesis, but long-term randomized controlled studies are also needed to evaluate the changes caused by diabetes-related complications on sexual dysfunction over time.
